# Gender and Age Trends in HIV Incidence in Turkey between 1990 and 2021: Joinpoint and Age–Period–Cohort Analyses

**DOI:** 10.3390/medicina60081357

**Published:** 2024-08-21

**Authors:** Okan Derin

**Affiliations:** 1 Epidemiology PhD Program, Graduate School of Health Sciences, İstanbul Medipol University, 34810 İstanbul, Türkiye; okan.derin@std.medipol.edu.tr; 2 Infectious Diseases and Clinical Microbiology Clinic, İstanbul Şişli Hamidiye Etfal Training and Research Hospital, 34396 İstanbul, Türkiye

**Keywords:** HIV, APC analysis, joinpoint regression, Türkiye, gender equity, sustainable development

## Abstract

*Background and Objectives:* Despite a global decrease in HIV incidence, recent trends in Türkiye indicate a concerning rise, particularly among younger populations and women. This study investigates the local and regional dynamics influencing these trends using advanced epidemiological methodologies. *Material and Methods:* Utilizing Age–period–cohort analysis and joinpoint regression, we analysed HIV incidence and prevalence data from the Global Burden of Disease study for Türkiye. These methods allowed for a detailed examination of changes over time, identifying specific age groups and periods with significant shifts in incidence rates. *Results:* Key findings include a 13.03% increase in annual percentage change among males aged 15–19 and an 11.37% increase among females in the same age group. Additionally, the incidence rates among females have shown a significant rise after 2008. *Conclusions:* The rising HIV incidence in Türkiye reflects complex socio-economic, cultural, and biological factors, with significant increases among young people and women. Addressing these challenges requires targeted interventions, comprehensive educational programs, and inclusive healthcare services to align with global efforts and commitments. The study underscores the importance of incorporating young people in decision-making processes to effectively combat HIV in Türkiye.

## 1. Introduction

The global HIV/AIDS epidemic has undergone significant changes over the past few decades. While HIV was once a major cause for concern, advancements in treatment have transformed it into a manageable chronic condition [1,2]. Despite this progress, the impact of HIV on society and the economy remains substantial. Globally, the HIV incidence rate has decreased by 59% since 1995, yet approximately 0.7% of adults aged 15–49 were still living with HIV in 2022. The prevalence is higher among specific groups, such as men who have sex with men (8%), transgender people (10%), and people who inject drugs (5%). To address this, UNAIDS has set targets for testing and treatment, known as 95-95-95, with a focus on these key populations [3].

The first presence of AIDS was documented in 1985 in Türkiye, and since then, concerted efforts have been undertaken in collaboration with the international community to combat HIV [4]. Türkiye, situated at the crossroads of Eastern Europe and the Middle East, is experiencing a complex interplay of socioeconomic, cultural, and biological factors that may be contributing to the increasing HIV burden, distinguishing the global declining trend. Rapid societal changes, evolving healthcare infrastructure, and significant migration patterns have all shaped the country’s HIV landscape. Moreover, the persistent stigma associated with HIV/AIDS, particularly among vulnerable populations, continues to hinder prevention and treatment efforts. Over the past decades, Türkiye has witnessed significant shifts in societal attitudes, healthcare infrastructure, and migration patterns, all of which can exert profound influences on HIV epidemiology. A UNFPA report (United Nations Population Fund) underscores that Türkiye presently has a young population, constituting one-fifth of the total. While the proportion is anticipated to decrease by 2050, the absolute number of young individuals is projected to rise. Furthermore, there is an inadequacy in the public provision of sexual and reproductive health services in Türkiye [5].

The objective of this study is to identify the key factors that have led to a significant change in incidence rates over time in Turkey. In addition, the study will examine the complex interplay between age, period, and cohort effects. We hypothesize that socio-economic, cultural, and biological factors significantly influence these trends. Within this context, the age–period–cohort analysis emerges as a powerful tool, offering a nuanced perspective on how diseases evolve over time [6,7]. As the global community strives towards the United Nations’ ambitious goal of ending the AIDS epidemic by 2030 [8], as a part of sustainable development goal 3, a granular understanding of regional dynamics is essential [9].

In our study, the main aim is to delineate breakpoints signifying the change in incidence over time using joinpoint analysis, and to explore the complex interplay between age, period, and cohort effects.

## 2. Materials and Methods

A comprehensive framework presented by Shri et al. [10], which is an important foundation for our research efforts, has been carefully drawn from the methodology applied in this study.

The Global Burden of Disease (GBD) study assesses 369 diseases and injuries across 204 countries, considering both sexes. It provides crucial health metrics, including incidence, prevalence, mortality, years of life lost (YLLs), years lived with disability (YLDs), and disability-adjusted life-years (DALYs). Data is sourced from various reliable channels, including censuses, household surveys, civil registration and vital statistics, disease registries, health service use, air pollution monitors, satellite imaging, disease notifications, and published scientific articles. Complex statistical models, such as the cause of death ensemble model and spatiotemporal Gaussian process regression, are employed to calculate cause-specific death rates and fractions. The socio-demographic index (SDI) contextualizes the results. Uncertainty intervals (UIs) were generated to account for variations in the data. This dataset serves as a pivotal resource for understanding global health trends and guiding evidence-based interventions [11].

The data on incidence, prevalence, and mortality for Türkiye were obtained from the Global Health Data Exchange (GHDx) online query tool of the GBD study. This tool is publicly accessible and maintained by the Institute for Health Metrics and Evaluation (IHME) [12]. The obtained data contains prevalence, incidence, and death rates specific to Türkiye, categorized by sex (female, male, and both sexes), and presented per 100,000 population. The dataset was further stratified by age, utilizing intervals of 5 years, with a minimum age of 15 and a maximum of 70+, yielding 12 distinct age groups. UIs were used to calculate standard errors at a 95% confidence level: [$\frac{upper\;bound-lower\;bound}{2*Z}]$. Additionally, the cohort (C) variable was calculated by subtracting the upper value of the age group (A) from the relevant period (P) [C=P−A] [13].

Both joint-point regression analysis and age–period–cohort analysis were used to assess trends in HIV incidence and prevalence in Türkiye. Joinpoint regression analysis, conducted using the joinpoint regression program version 5.0.2 from the Statistical Research and Applications Branch of the National Cancer Institute [14], was used to identify potential changes in age-standardized incidence rates (ASIRs) among the overall population as well as within sex-specific subgroups across all age groups [15]. Joinpoint regression analysis autonomously determines the location and number of joinpoints within the model, rather than relying on predetermined specifications. The joinpoint software 5.2.0.0 (April 2024) is used to determine the number of segments necessary to characterize a trend, where the segments begin and end, and the annual percent change (APC) [16] for each segment. APC represents the yearly rate of change in the incidence rate within each identified segment. It is calculated by fitting a linear regression model to the logarithm of the incidence rates over time. Mathematically, the APC for a given segment can be expressed as:

The regression model: $\log\left(R_{y}\right)=b_{0}+b_{1\;}y$

$$APC_{\Delta \left[\left(y+1\right),y\right]}=\left[\frac{\left(R_{y+1}-R_{y}\right)}{R_{y}}\right]\times 100=\;\ldots \;=\left(e^{b1}-1\right)\times 100$$

where $\log\left(R_{y}\right)$ is the natural log of the rate in year y.

In addition to the APC, the report also presents the average annual percent change (AAPC), which is a summary measure of the trend over a fixed, pre-specified interval. The AAPC represents the weighted average of the APCs for each segment within the interval, providing a single summary measure that reflects the overall trend. This metric is particularly useful for comparing the most recent trend across distinct groups when the final joinpoint segments are not directly comparable [17,18]. If we denote $b_{i}$ as the slope coefficient for the $\;i^{th}$ segment with i indexing the segments in the desired range of years and $w_{i}$ as the length of each segment in the range of years, then: $APC_{i}=\{\exp\left(b_{i}\right)-1\}\times 100$. The AAPC is calculated using the formula:

$$AAPC=\left\{\exp\left(\frac{\sum w_{i}b_{i}}{\sum w_{i}}\right)-1\;\right\}\times 100$$

For our analysis, HIV incidence data were classified into structured age groups and time periods, both segmented into five-year intervals. We utilized pre-calculated age-standardized incidence rates (ASIRs) directly from the Institute for Health Metrics and Evaluation (IHME) database, where age-standardization is applied to each data point independently [19]. These ASIRs are derived using the Global Burden of Disease (GBD) standard population as the reference, ensuring comparability across different populations and time periods [11].

To conduct the age–period–cohort analysis of HIV incidence rates in Türkiye, we utilized the “APCI” package in R [20,21,22], which implements the age–period–cohort-interaction (APC-I) model [23]. This model enhances traditional age–period–cohort analyses by integrating interaction terms between age and period to effectively capture cohort effects. The APC-I model is advantageous as it addresses the inherent identification problem in traditional age–period–cohort analysis models by considering these interactions, thus allowing a nuanced exploration of how cohort-specific changes influence HIV incidence rates over time. The model estimates incidence coefficients, which quantify the strength and direction of the association between each of the three factors (age, period, and cohort) and the incidence rates. These coefficients provide insight into how the incidence of HIV changes across different age groups, time periods, and birth cohorts and are expressed along with their standard errors (SE) in the results. The APC model allows us to disentangle the effects of age, period, and cohort on the incidence rates. For instance, a positive age coefficient indicates an increase in HIV incidence with age, while a negative cohort coefficient suggests that individuals born in later cohorts have a lower risk compared to those born in earlier cohorts.

## 3. Results

### 3.1. Comparison of Age-Standardized Incidence Trends in Türkiye with Global Averages

**Figure 1:**
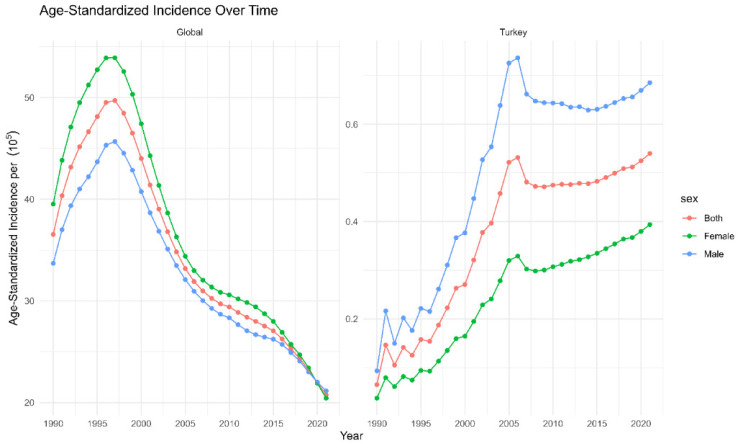
Age-standardized incidence rates (ASIRs) [19] of HIV/AIDS from 1990 to 2021 in Turkey and globally. Note the significant rise in ASIRs among Turkish females post-2008, indicated by a marked increase in the corresponding trend lines.

In both global and Turkish contexts, Figure 1 depicts the age-standardized incidence of HIV/AIDS over the last three decades (1990–2021). In Türkiye, ASIRs increased slightly until 1997, which corresponds to the global peak incidence for both sexes. Afterwards, there was a rapid increase in Türkiye between 1997 and 2005, even though global ASIRs tended to decrease during the same period. The highest ASIRs in Türkiye for males (0.77; UI = 0.64, 0.88), females (0.38; UI = 0.29, 0.41), and both sexes (0.55, UI = 0.48, 0.64) were observed in 2006. After 2006, ASIRs of HIV in Türkiye decreased until 2007, but started to increase in both sexes afterwards, despite the decreasing global trend.

### 3.2. Joinpoint Analysis

**Figure 2:**
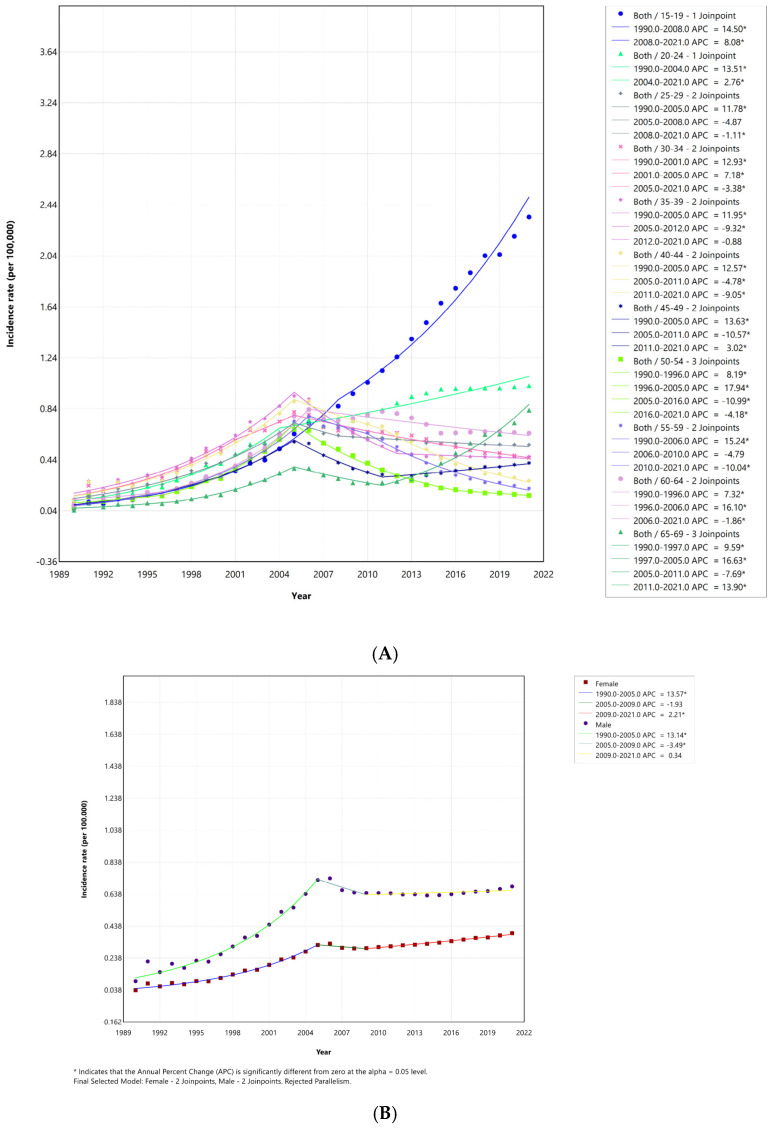
(**A**) Age group-stratified joinpoint regression analysis of incidence rates between 1990 and 2021 in Türkiye; APC: annual percentage change; (*) indicates *p* < 0.05. (**B**) Gender-stratified joinpoint regression analysis of incidence rates between 1990 and 2021 in Türkiye; APC: annual percentage change; (*) indicates *p* < 0.05.

Table 1 provides AAPC in HIV incidence rates in Türkiye from 1990 to 2021, stratified by age group and gender. The analysis utilized joinpoint regression to identify significant changes in trends over time. The age-standardized AAPC of 6.19% indicates that HIV incidence has been generally increasing in Türkiye over the study period. Females experienced a higher overall AAPC (6.98%) compared to males (5.81%), suggesting a faster increase in HIV incidence among women. The most striking increases were observed in younger age groups. The 15–19 age group had the highest AAPC (11.77%), followed by the 20–24 age group (7.49%). In almost all age groups, females had a higher AAPC than males. This difference was particularly pronounced in the 30–34 age group. Details of the joinpoint analysis of ASIRs are shown in Figure 2. The ASIR for women increased significantly after 2008, whereas the ASIR for men decreased.

### 3.3. Age–Period–Cohort (APC) Analysis

Table 2 in our study provides a detailed summary of age, period, and cohort effects on incidence coefficients across genders. This comprehensive display categorizes the effects into three distinct sections:

#### 3.3.1. Age Group Effects

The change in incidence coefficients for various age groups, highlighting significant variances across both genders, females, and males. Younger age groups (15–19 and 20–24) show positive coefficients, particularly notable in females with coefficients like 0.461 (0.016) ***, indicating a significantly higher incidence rate. In contrast, older age groups (45–49 and 50–54) generally exhibit negative coefficients, suggesting a decrease in incidence rates as age increases.

#### 3.3.2. Period Effects

Reflecting changes over distinct time periods, this part shows how incidence coefficients have evolved from 1990 to 2021. Early periods (1990–1994 and 1995–1999) record predominantly negative coefficients across all groups, pointing to a decrease in incidence rates. However, more recent periods (2005–2021) reveal a reversal of this trend with positive coefficients, indicating an increase in the incidence rate.

#### 3.3.3. Cohort Group Effects

The coefficients here highlight how specific cohorts are differently affected over their lifetimes. Notably, earlier cohorts, like those born between 1971 and 1975, show significantly negative effects. Conversely, more recent cohorts (1991–1995, 1996–2000) show highly positive coefficients.

**Figure 3:**
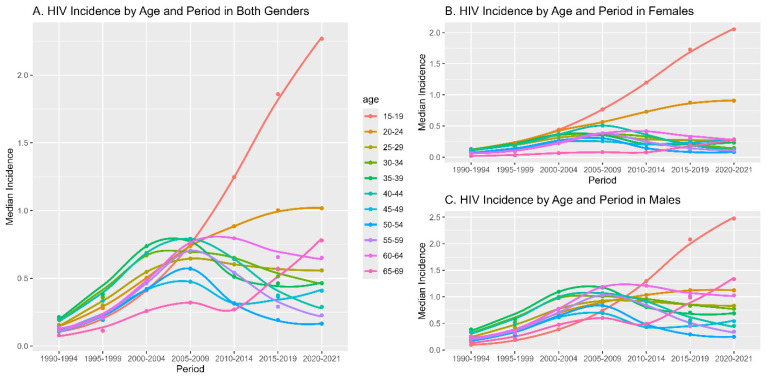
HIV incidence by age and period in Türkiye.

Figure 3 illustrates the median HIV incidence by age group and period, disaggregated into three categories: both genders combined, females only, and males only. Each line represents a different age group, indicated by distinct colors, spanning from ages 15 to over 65. The *x*-axis represents consecutive time periods from 1990 to 2021, divided into five-year intervals (except for the last period, which consists of 2 years).

For all combined (Graph A), most age groups show an increase in median HIV incidence up to the period 2005–2009, followed by a stabilization or slight decline in the subsequent periods, except for the youngest age group (15–19), which spikes dramatically in the latest period (2015–2021).

In the breakdown by gender (Graphs B for females and C for males), similar patterns are observable, with some variations between the genders. For females, the rise in incidence appears more pronounced in the latest period across almost all age groups, while in males, the increase is more gradual but shows a sharper rise in the youngest age group in the last period.

## 4. Discussion

### 4.1. Overview of Global and Local Trends

Our research primarily reveals a recent rise in HIV incidence in Türkiye, especially among younger individuals and women, despite a global decline in overall rates (Figure 1).

The global number of new HIV infections has declined by 39% since 2010 [24,25]; however, the incidence of HIV has increased in Eastern Europe (33%), the Middle East, and North Africa (27%), and Latin America (11%) [25]. Notably, Türkiye, situated within Eastern Europe and the Middle East, is experiencing an increase in new HIV cases. This regional pattern suggests that certain factors contributing to the rise in HIV incidence in Türkiye may be similar to those observed in Eastern European and Middle Eastern contexts. Global efforts to achieve the sustainable development goals (SDGs) and the 2030 UNAIDS targets have resulted in more accessible antiretroviral therapy (ART), a global decrease in new HIV/AIDS cases, particularly in sub-Saharan Africa, and lower mortality rates from HIV/AIDS. However, there appears to be a lack of preventative measures adoption in countries within the aforementioned regions. A recent review [25] highlighted the challenges hindering the adoption of HIV prevention measures in different regions. For Eastern Europe, the key issues include insufficient access to sterile injecting equipment, the unavailability of opioid substitution therapy, stigma, the criminalization of key populations leading to violence, and armed conflicts. In the Middle East, the primary challenges are reaching key populations, stigma, and the criminalization of these groups in some countries.

However, joinpoint analysis indicates that there has been a greater increase in HIV incidence among males (AAPC = 12) than females (AAPC = 11) over the 31-year period (Table 1). Upon closer examination of the data for the most recent decade, it becomes evident that there has been a significant increase in HIV incidence among females (Figure 1). We observed an age-standardized average annual percentage change (AAPC) of 6.19 [6.18, 6.92] across both genders between 1990–2021 (Table 1). This increase was more pronounced in younger age groups (15–24) and females (Figure 2). This mirrors the global trend where young people (15–24 years) account for a substantial proportion of new infections despite overall progress in reducing HIV incidence [26].

The cohort deviation observed in the 1946–1956 groups post-2000 (Table 2 and Figure 3) may be attributed to socio-economic transitions and changes in healthcare accessibility. Joinpoint analysis depicted that people aged 65–69 had a high annual percentage change after 2011; however, this was not presented in the APC-I model. Although a general increasing trend has been reported in women over fifty in developed countries [27,28], we were unable to observe this trend in Türkiye. Low perceptions of HIV risk and low condom use have been reported among women over 50 years old in China [29]. Further rigorous studies are needed to draw reliable conclusions about the over-50 age group.

### 4.2. Stigma/Criminalization of Key Populations and Gender Disparities

As a prevention strategy, governments implemented treatment-as-prevention (TasP) policy and accepted widely. To ensure the success of TasP, it is crucial to tackle the significant challenges posed by stigma, which often leads to reluctance in participating in screening and treatment [30].

A study conducted in Türkiye [31], which included men who have sex with men (MSM), transgender individuals, women, sex workers, and intravenous drug users, along with others, revealed that 23% of the participants experienced stigma, and 30% were subjected to violence. The study concluded overall rates of stigma and violence were lower than in other European countries, but were higher in the vulnerable groups. A non-governmental organization (NGO) has published a series of reports [32] indicating that, in Türkiye, stigma often results from the mandatory disclosure of medical records, especially among young adults who are in educational settings or at the initial stages of their careers. This stigma has a significant impact on individuals living with HIV, preventing them from initiating or adhering to treatment and causing a range of social and psychological effects.

According to UNAIDS’ “Youth and HIV report”, young key populations, including gay and bisexual men, transgender individuals, young sex workers, and drug injectors, face a significantly elevated risk of HIV infection and human rights abuses. A study from the United States of America revealed that transgender women in Florida face significant barriers to accessing and using PrEP, including systemic oppression, discrimination, and lack of representation, which contributse to a syndemic of health challenges [33]. Studies show that female sex workers and drug injectors are 13 and 21 times more likely to contract HIV, respectively. Similarly, gay and bisexual men are 27 times, and transgender women are 12 times more likely to acquire HIV than the general population [34]. For Türkiye to effectively combat the rising incidence of HIV among its vulnerable young populations, it is imperative to implement tailored prevention measures and ensure the availability of inclusive healthcare services as free anonymous HIV/STD screening. This approach should focus on the specific needs of key groups, including gay and bisexual men, transgender individuals, young sex workers, and drug injectors, who are at significantly higher risk. The development and implementation of targeted health interventions that address both the medical and social challenges faced by young population, in conjunction with the fostering of an environment of non-discrimination and support within healthcare settings, represent crucial steps in reducing the prevalence of HIV and improving overall public health outcomes in the country.

Non-adherence to treatment can undermine the effectiveness of treatment-as-prevention (TasP) strategies. A study conducted in Türkiye found that non-adherence to antiretroviral therapy (ART) is associated with being female [35]. The UNAIDS report indicates a high burden of HIV among young women globally, consistent with our findings in Türkiye that show a significant rise among females [26]. The increasing incidence of HIV among women in Türkiye reflects a complex interplay of socio-economic, cultural, and biological factors. Biologically, women are more susceptible to HIV due to physiological factors. Many women face social and economic challenges, including limited access to education [36] and increased stigma/violence [37,38] which are crucial for effective prevention and treatment of HIV. Cultural norms may also hinder women’s ability to negotiate safer sex practices and seek timely medical help [38,39]. Efforts by organizations like UN Women in Türkiye, which focus on enhancing gender equality and empowering women, are vital. They help address underlying issues, such as gender-based violence and educational inequities. Nevertheless, there is still a need for targeted HIV prevention programs that specifically address the unique vulnerabilities of women. Collaborative approaches involving the government, local NGOs, and international bodies are essential to creating sustainable changes in public health strategies that can reduce the incidence of HIV among women in Türkiye.

Sex work in Türkiye has been regulated through state-operated brothels, where bi-monthly STD screenings and mandatory condom use during intercourse are mandated by law. Despite these regulations, there were notable gaps in adherence to these practices. Recent shifts in government policy have led to the closure of these legal facilities, simultaneously with significant sociocultural and digital (“Cyber dating and hookup culture, Sex workers’ platforms, websites, and forums”, etc.) [40] transformations, catalyzing a rise in unregistered sex work. Currently, it is estimated that unregistered sex workers vastly outnumber their registered counterparts, with figures at 85,000 compared to 15,000, respectively [41,42]. In a study conducted in Romania, a relationship was found between CD4 cell count, HIV viral load, age at first sexual intercourse, number of sexual partners, and HPV coinfection in women living with HIV [43]. In these unregulated environments, STD prevention measures like more accessible STD screenings and condom use pose increased health risks and increase HIV incidence. The establishment of anonymous testing centers and the provision of STD consultations could serve as critical interventions to mitigate these emerging public health challenges [44].

### 4.3. Cultural and Social Beliefs

Türkiye is often characterized as a secular country with a predominantly Muslim population. Despite the secular framework of the state, most of the Turkish population practices Islam, making it a predominantly Muslim country culturally and religiously. HIV prevalence is lower in Islamic countries particularly in MENA (Middle East and North Africa) countries due to strict societal and legal prohibitions against behaviors, such as sexual promiscuity, same-gender acts, and intravenous drug use, which are major risk factors for HIV transmission [45]. In some Islamic countries, these acts are punishable by severe penalties, including death [46]. More recent publications refute the correlation between increased religious behavior and lower HIV/STD prevalence [47,48]. A recent systematic review suggested increasing HIV prevalence in MSMs living in several MENA countries and emphasized importance of HIV surveillance and access to HIV testing, prevention, and treatment services [49].

In fact, as a secular country, Türkiye is considered to have cultural characteristics distinctly influenced by religion, setting it apart significantly from the traditional lifestyles seen in other MENA countries, which are predominantly shaped by Arab or Iranian influences [50]. However, as mentioned earlier, discrimination, stigma, and violence are prevalent issues faced by people living with HIV (PLWH). An intriguing case report from Türkiye highlights the significant challenges faced by gay people, emphasizing the profound influence of cultural and religious norms in the country [51]. Limited reproductive health knowledge impedes women’s access to contraception and other reproductive services in Muslim countries [52]. Two reports have suggested that Islamic countries have an opportunity to combat HIV/AIDS by challenging taboos and fostering collaboration with Islamic scholars and leaders [47,48]. Given the examples provided, enhancing interventions targeting vulnerable groups could play a pivotal role in reversing the increasing incidence of HIV/AIDS in Türkiye.

### 4.4. Mobility and Migration

Since 2011, Türkiye has grappled with mass migration and irregular human mobility arising from the Syrian crisis. Over the past several years, Türkiye has consistently ranked as the country with the highest number of refugees and asylum seekers worldwide [53]. An article from Poland pointed out that the entry of refugees into the country after the Ukraine war may be associated with an increase in HIV incidence [54]. There is no detailed and precise report available discussing HIV epidemiology of the refugees in Türkiye. A single-center study using convenience sampling reported no HIV seropositivity among refugees in Türkiye [55]. A study conducted at the Greek–Turkish border in Greece found a 0.3% HIV seropositivity rate among refugees [56]. The prevalence of HIV in Türkiye is similar to these findings. The study concluded that comprehensive surveillance of irregular migrants is essential to prevent the spread of communicable diseases and to enhance healthcare services for this group. As a vulnerable population (e.g., exposed illegal sexual networks, increased substance abuse, and limited access to healthcare services), irregular immigrants would benefit from thorough surveillance to prevent transmissible diseases, including HIV, and to access more qualified healthcare services. For the sake of the paper, the central inquiry is as follows: Does the upward trend among younger individuals and particularly females, correlate with the refugee crisis? While sex work is legally regulated, sex trafficking is considered a crime punishable by 8 to 10 years in prison in Türkiye [57]. Yet, females and LGBT refugees are considered vulnerable for human trafficking and sex slavery in the same report. Those people have limited access to health-care services for refugees [58]. Human trafficking and sex slavery might contribute to increasing HIV incidence among youth, especially females. Key risks may include high-risk behavior exposure, lack of access to prevention and healthcare services, stigma and discrimination, and limited autonomy and agency. Understanding these intersectional and multifaceted risks is crucial for developing effective interventions and support mechanisms to address the HIV-related needs of individuals affected by human trafficking and sex slavery.

### 4.5. Educational and Preventive Measures

Education level, especially among youth and females, has been shown to be strongly associated with overall health status [59]. UNAIDS emphasizes the low level of HIV knowledge among youth worldwide, with only one-in-three young people having accurate HIV prevention knowledge [34]. Comprehensive school-based sex education programs, adapted from successful models and incorporating various school and community elements, had the most significant effect on altering HIV-related behaviors in lower- and middle-income countries [60,61,62]. Between 1974 and 2003, sexual and reproductive education was provided by both governmental and non-governmental organizations in Türkiye [63]. However, studies assessing the knowledge of sexually transmitted diseases among university students have revealed alarmingly low rates [64,65,66]. Establishing well-designed educational initiatives tailored to gender, age, and cultural factors in Türkiye could play a pivotal role in curbing the increasing rate of HIV infections among young individuals. Ensuring active involvement of young people in decision-making regarding HIV prevention aligns with UNAIDS recommendations.

## 5. Conclusions

In conclusion, our analysis underscores the concerning rise in HIV incidence among younger individuals and women in Türkiye, contrasting with the global trend of declining rates. This increase mirrors patterns observed in Eastern Europe and the Middle East, indicating shared contributing factors. Stigma, particularly prevalent among vulnerable groups, poses a significant barrier to effective prevention and treatment efforts. Targeted interventions addressing the unique needs of key populations, such as young sex workers and drug injectors, alongside broader societal challenges like cultural norms and access to education, are imperative. Additionally, considering the implications of mass migration on HIV epidemiology underscores the need for comprehensive surveillance and support mechanisms for vulnerable populations. To effectively combat the rising HIV incidence in Turkey, it is crucial to implement targeted interventions, enhance educational programs, and ensure comprehensive healthcare services. Prioritizing the inclusion of youth and women in these initiatives will be pivotal.

## Figures and Tables

**Table 1:** Average annual percentage change (AAPC) of HIV incidence in Türkiye by age and gender from 1990 to 2021 using the joinpoint regression analysis.

	Average Annual Percentage Change [95% CI]		
Age	Both	Female	Male
15–19	11.77 [11.14, 12.39]	11.04 [10.43, 11.65]	12.48 [11.83, 13.13]
20–24	7.49 [6.85, 8.12]	7.96 [7.31, 8.62]	7.18 [6.54, 7.83]
25–29	4.54 [3.41, 5.68]	4.13 [3.36, 4.90]	4.58 [4.04, 5.12]
30–34	3.50 [2.47, 4.53]	1.50 [0.25, 2.76]	3.75 [3.18, 4.33]
35–39	3.04 [2.27, 3.81]	3.60 [2.73, 4.48]	2.95 [2.16, 3.76]
40–44	1.73 [0.83, 2.64]	1.02 [−0.06, 2.12]	2.27 [1.62, 2.92]
45–49	5.11 [4.11, 6.11]	5.58 [4.44, 6.72]	3.95 [2.56, 5.35]
50–54	1.50 [0.29, 2.73]	1.09 [−0.20, 2.40]	1.57 [−0.04, 3.20]
55–59	2.98 [1.74, 4.23]	2.25 [0.85, 3.68]	2.18 [0.73, 3.66]
60–64	5.41 [4.03, 6.81]	5.70 [4.14, 7.30]	5.36 [4.13, 6.60]
65–69	9.07 [7.40, 10.78]	11.20 [9.23, 13.20]	8.67 [7.00, 10.37]
70–74	0.85 [−1.08, 2.82]	0.31 [−1.73, 2.40]	1.05 [−0.92, 3.07]
75–79	−4.81 [−8.64, −0.81]	−5.81 [−10.06, −1.35]	−4.09 [−9.03, 1.12]
Age-standardized	6.19 [5.56, 6.83]	6.98 [6.33, 7.64]	5.81 [5.17, 6.45]

**Table 2:** Summary of age, period, and cohort effects on incidence coefficients across genders.

	Both Coefficients (SE)	Female Coefficients (SE)	Male Coefficients (SE)
Age group effects			
15–19	0.490 (0.016) ***	0.626 (0.015) ***	0.353 (0.019) ***
20–24	0.157(0.012) ***	0.240 (0.007) ***	0.073 (0.010) ***
25–29	−0.018(0.021)	−0.051 (0.005) ***	0.012 (0.233)
30–34	0.008(0.024)	−0.063 (0.005) ***	0.077 (0.013) ***
35–39	0.009(0.024)	−0.067 (0.007) ***	0.082 (0.017) ***
40–44	−0.026(0.019)	−0.047 (0.007) ***	−0.007 (0.015)
45–49	−0.167(0.014) ***	−0.105 (0.004) ***	−0.231 (0.012) ***
50–54	−0.210(0.014) ***	−0.153 (0.006) ***	−0.267 (0.013) ***
55–59	−0.130(0.018) ***	−0.124 (0.005) ***	−0.134 (0.014) ***
60–64	0.037(0.027)	−0.054 (0.005) ***	0.133 (0.013) ***
65–69	−0.151(0.029) ***	−0.201 (0.004) ***	−0.091 (0.015) ***
Period effects			
1990–1994	−0.347(0.008) ***	−0.221 (0.003) ***	−0.4750.008 ***
1995–1999	−0.234(0.010) ***	−0.153 (0.005) ***	−0.3150.011 ***
2000–2004	0.001(0.016)	−0.017 (0.007) *	0.0180.016
2005–2009	0.175(0.018) ***	0.094 (0.006) ***	0.2550.012 ***
2010–2014	0.117(0.016) ***	0.0740.006 ***	0.1600.011 ***
2015–2019	0.126(0.015) ***	0.0960.006 ***	0.1550.009 ***
2020–2021	0.163(0.024) ***	0.1260.004 ***	0.2000.008 ***
Cohort group effects			
1921–1925	0.076 (0.029) **	0.135 (0.005) ***	0.011(0.020)
1926–1930	−0.018 (0.017)	0.054 (0.005) ***	−0.095 (0.017) ***
1931–1935	−0.020 (0.013)	0.024 (0.005) ***	−0.065 (0.020) **
1936–1940	0.005 (0.013)	0.019 (0.007) **	−0.006 (0.020)
1941–1945	0.058 (0.013) ***	0.043 (0.006) ***	0.075 (0.016) ***
1946–1950	0.125 (0.013) ***	0.074 (0.006) ***	0.178 (0.016) ***
1951–1955	0.101 (0.017) ***	0.039 (0.006) ***	0.163 (0.016) ***
1956–1960	−0.002 (0.011)	−0.008 (0.006)	0.002 (0.015)
1961–1965	−0.023 (0.010) *	0.005 (0.005)	−0.052 (0.014) ***
1966–1970	−0.030 (0.012) **	−0.030 (0.007) ***	−0.030 (0.014) *
1971–1975	−0.144 (0.010) ***	−0.141 (0.006) ***	−0.147 (0.012) ***
1976–1980	−0.195 (0.010) ***	−0.171 (0.007) ***	−0.218 (0.010) ***
1981–1985	−0.203 (0.012) ***	−0.175 (0.008) ***	−0.231 (0.010) ***
1986–1990	−0.132 (0.015) ***	−0.105 (0.011) ***	−0.159 (0.016) ***
1991–1995	0.095 (0.018) ***	0.103 (0.017) ***	0.086 (0.026) ***
1996–2000	0.483 (0.021) ***	0.456 (0.027) ***	0.509 (0.029) ***
2001–2006	1.111 (0.031) ***	0.994 (0.039) ***	1.226 (0.050) ***

Table presents coefficients and standard errors for age, period, and cohort effects across both genders, females, and males. Entries include significance levels indicated by asterisks: *** (*p* < 0.001), ** (*p* < 0.01), and * (*p* < 0.05).

## Data Availability

Online public data were used in this study.
